# Forest canopy interactions with aerosols: important considerations in approaching future impacts and climate management

**DOI:** 10.1111/nph.70636

**Published:** 2025-10-09

**Authors:** Maxime Durand, Anna Lintunen, Ekaterina Ezhova

**Affiliations:** ^1^ Organismal and Evolutionary Biology (OEB), Viikki Plant Science Centre (ViPS), Faculty of Biological and Environmental Sciences University of Helsinki Helsinki Finland; ^2^ Institute for Atmospheric and Earth System Research (INAR)/Forest Sciences, Faculty of Agriculture and Forestry, Viikki Plant Science Centre (ViPS) University of Helsinki Helsinki Finland; ^3^ Institute for Atmospheric and Earth System Research (INAR)/Physics University of Helsinki, Faculty of Science Helsinki Finland

**Keywords:** aerosol particles, carbon and water cycles, climate change, diffuse light, forests, geoengineering, spectral composition, wildfires

## Abstract

Aerosols influence forest ecosystems through changes in radiation and climate affecting plant physiology and structure. Conversely, forests also contribute to aerosol formation. They emit primary aerosol particles and volatile organic compounds, which promote secondary organic aerosol formation in the atmosphere. This forest–aerosol coupling is highly dynamic, influenced by temperature, radiation, humidity, and trace gases. Wildfires add further complexity via smoke plumes altering radiation and ecosystem functioning, tropospheric ozone levels and stratospheric chemistry. Aerosols modify the quantity, directionality, and composition of solar radiation. The type of diffuse light produced by aerosol particles is however strongly dissimilar to the one produced under clouds, and the relevance of the traditional diffuse/direct binary paradigm is discussed. Therefore, potential benefits from increased diffuse radiation are contingent on aerosol load, canopy structure, and prevailing environmental conditions. Beyond photosynthetic responses, aerosols alter forest water‐use efficiency and microclimate, yet their long‐term effects on plant development, architecture, and community composition remain poorly understood. This review highlights significant knowledge gaps and recent advances in understanding aerosol–forest interactions across temporal and spatial scales. We underline the urgent need for improved experiments with realistic diffuse shading, extensive *in situ* observations, mechanistic model intercomparison, and global validation to guide future research and policy.

**Table:** 

	Contents	
	Summary	114
I.	Introduction	114
II.	Forests' role in aerosol production	115
III.	Short‐term aerosol effects on forests	116
IV.	Long‐term aerosol effects on forests	117
V.	Conclusion	119
	Acknowledgements	119
	References	120

## I. Introduction

Aerosols are tiny particles suspended in the atmosphere. They scatter and absorb radiation, directly affecting its quantity and quality and consequently air temperature. They can also indirectly affect the climate by acting as cloud condensation and ice nuclei (see Lohmann & Feichter, 2005). Although aerosol emissions in many parts of the globe have started to decrease since 2014 (Quaas *et al*., 2022), future trends are still uncertain because of increased temperatures, land‐use changes (Shi *et al*., 2025), wildfires (Katich *et al*., 2023), continued industrial pollution in Asia, future climate policies, and unpredictable political trends. Moreover, climate interventions that propose to inject aerosol particles into the stratosphere are now seriously being considered (Lee *et al*., 2023), accelerating the need to comprehensively decipher how aerosols interact with natural ecosystems in the short‐ and long‐term.

**Fig. 1:**
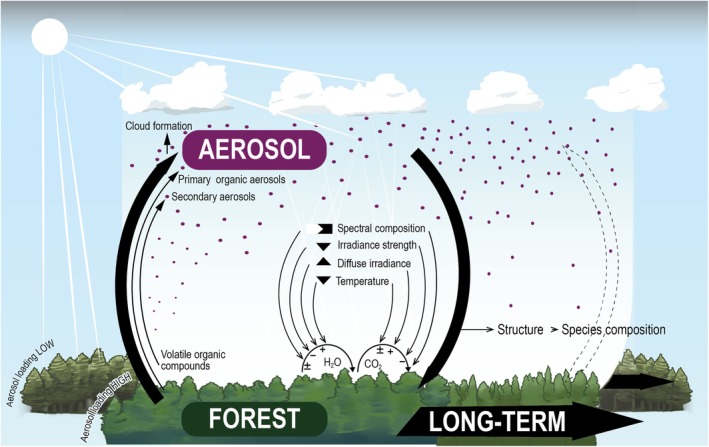
Summary representation of interactions between aerosol particles and vegetation. Aerosols affect forests (large descending arrow) by modifying irradiance strength, composition, diffuseness, and air temperature inducing changes in carbon and water fluxes. In turn, forests affect aerosols composition and amounts (large ascending arrow) by releasing volative organic compounds that can lead to aerosol formation. These effects are suspected to alter forest structure and species composition in the long term.

Forests occupy 31% of the global land surface, yet processes at the leaf, plant, and ecosystem scale governing how forests respond to and affect aerosol loading are not fully understood. In this short review focused on the latest research, we aim to provide an integrated overview of the diverse interactions between aerosols and forests, bringing together atmospheric processes, canopy responses, and regional dynamics that are often considered in isolation. We outline how forests can contribute to aerosol populations, and conversely, the specific effect of aerosols on forests in the short‐ and long‐term (Fig. 1). By surveying these many interconnected facets, each warranting its own full‐length review, we seek to identify priority directions for future research.

## II. Forests' role in aerosol production

Forest ecosystems emit primary aerosol particles (e.g. pollen and fungal spores) and biogenic volatile organic compounds (BVOCs). BVOCs are oxidised in the atmosphere to produce organic vapours, contributing to the formation and growth of secondary aerosol particles via gas‐to‐particle conversion (or new particle formation, NPF). About half of the global cloud condensation nuclei originate from secondary aerosol formation (Merikanto *et al*., 2009). Another source of aerosol particles is the biomass burning of forests, discussed in Box 1.

Biogenic secondary organic aerosol (BSOA) dominates secondary aerosol formation: annual SOA fluxes contain about nine times more biogenic than anthropogenic secondary aerosol (Hallquist *et al*., 2009). Isoprene, emitted mainly by tropical forests, constitutes more than half of global BVOC emissions to the atmosphere (1000 TgC yr^−1^; Satake *et al*., 2024). Although it had long been assumed that isoprene does not contribute to SOA production (Lee *et al*., 2006), isoprene nitrates were recently found to contribute to particle formation in the troposphere (Curtius *et al*., 2024). Monoterpenes, emitted by coniferous tree species in boreal forests, are, on the other hand, smaller in quantity (one third of all emissions), but have a larger SOA yield, meaning they produce more SOA matter from the same amount of vapours (Lee *et al*., 2006). Monoterpenes and sesquiterpenes drive significant NPF in the atmospheric boundary layer (Artaxo *et al*., 2022). Although the feedback effect of BVOC emissions on forest productivity is difficult to isolate, early modelling results report that they could enhance global primary productivity by 1.23 Pg C yr^−1^ (Rap *et al*., 2018). Other authors reason that the true effect is likely to be smaller if accounting for modelling uncertainty, interactions between radiative, chemical, and precipitation impacts on productivity, and community dynamics altering species composition (Wang *et al*., 2019; Lund *et al*., 2021).

Future trends in BVOC emissions from forests are difficult to predict due to conflicting interacting processes. Emissions of BVOC increase strongly with temperature, but the effect of drought on BVOC emission is complex, depending on drought severity, plant species and tolerance, and BVOC compounds (Yang *et al*., 2025). Although reports show increases after 2 wk following drought onset (Fitzky *et al*., 2023), isoprene and monoterpene emissions are generally reduced under severe drought (Yang *et al*., 2025). NPF can also either increase or decline as BVOC oxidation, SOA formation, and yields are sensitive to temperature (Kürten *et al*., 2018); thus, the season when warming occurs could be a key factor. Moreover, decreasing anthropogenic emissions due to climate policies could also locally affect NPF, depending on background levels of biogenic and anthropogenic emissions. As an example, decreasing SO_2_ will decrease BSOA formation (Nascimento *et al*., 2021). This synergistic effect of biogenic–anthropogenic emissions for NPF is demonstrated by an increase in SOA formation in urban–forest areas (Ma *et al*., 2024), and in polluted air masses over Siberian forests (Garmash *et al*., 2024).

Overall, modelling studies reveal a 36% increase in BSOA production despite a 19% reduction in BVOC emissions compared to the preindustrial era because anthropogenic emissions are larger while vegetation cover is smaller (Zheng *et al*., 2023). Nevertheless, future projections are scenario‐dependent: for example, Community Earth System Model (CESM) 2.1 simulations using explicit chemistry predict global changes of isoprene‐derived SOA concentration in the 2090s of 45–278% relative to present conditions (Jo *et al*., 2021); notably, SOA yield from isoprene varies by 50% depending on the scenario's sulfur emissions. Generally, CMIP6 models show that only strong mitigation (SSP1‐2.6) reduces annual concentration in particles, while weak mitigation (SSP3‐7.0) still leads to further increases, except in Europe and North America (Turnock *et al*., 2020). However, even present‐day simulations with updated aerosol formation and growth schemes yield vastly different results: CESM 2.1 simulations with mechanistic description of highly oxygenated organic molecules (HOM) chemistry and new organic nucleation schemes show that global aerosol particle number burden increased by 39% compared to simulations with only inorganic nucleation, with the largest increase over Amazonia (Shao *et al*., 2024). Implementing mechanisms for NPF also showed strong regional variability (10–80%) in NPF contribution to particle number and cloud condensation nuclei (Zhao *et al*., 2024). Overall, models' representation of atmospheric chemistry, nucleation and growth schemes, aerosol properties and dynamics, as well as socio‐economic/emission scenarios underpin future outcomes.

Box 1 **When aerosols go up in smoke**

Although global wildfire activity has declined overall, mainly due to reduced fires in Africa (Chen *et al*., 2024), wildfires have increased significantly in many parts of the globe over the past two decades (Cunningham *et al*., 2024). While most ignitions are human‐caused (Balch *et al*., 2017), changing weather (temperatures, precipitation and humidity) and earlier snowmelt have created drier conditions that make any ignition far more likely to spread. Wildfire smoke significantly reduces solar irradiance at the ground, sometimes to only 30% of clear‐sky values (Kassianov *et al*., 2025), mainly via smoke production but also by promoting BVOC emissions (Ciccioli *et al*., 2014). Moderate smoke can also enhance plant productivity by increasing diffuse light, leading to improved photosynthesis (Hemes *et al*., 2020), and ecosystem CO_2_ uptake over several days (Rastogi *et al*., 2022). When smoke becomes denser (e.g. diffuse fractions over 80%), ecosystems can shift to net carbon sources (Lee *et al*., 2022), which may become increasingly common as future trends tend towards larger and more frequent fires (Behrer & Wang, 2024). Post‐fire, vegetation regeneration shows reduced BVOC emission, congruent with diminished vegetation cover and microbial activity in the soil (Zhang‐Turpeinen *et al*., 2021). Aerosol deposition from smoke will also impact forests in several ways. Ash and black carbon can stimulate or inhibit germination depending on the species (Reyes *et al*., 2015), and stimulate BVOC emission (Guo *et al*., 2024). A small amount of smoke deposition can also benefit plant growth by increasing nitrogen and carbon uptake, but plant growth is reduced at high levels of deposition (Zhan *et al*., 2025).

Plumes of smoke from intense fires develop into a special type of cloud, Pyrocumulonimbi, capable of injecting fire‐induced aerosol particles into the stratosphere and rivalling volcanic eruptions in scale. Recent observations found that pyrocumulonimbus accounts for up to 25% of the organic aerosol and black carbon currently in the stratosphere (Katich *et al*., 2023). These particles have 2–4 times stronger cooling effects than sulfate aerosols (Liu *et al*., 2022). They reduce precipitation (Zhu *et al*., 2025) and globally lower gross primary productivity by *c*. 1.5 Pg C per year due to combined effects on radiation and moisture (Xu *et al*., 2021). Wildfires also negatively impact vegetation by emitting precursors like NO_x_, CO, and VOCs that contribute to tropospheric ozone formation (Jaffe & Wigder, 2012). Recent evidence shows that large‐scale fires such as the 2019–2020 Australian wildfires have contributed up to 5% of stratospheric ozone depletion by releasing aerosol particles that facilitate ozone‐destructive reactions (Solomon *et al*., 2023).

## III. Short‐term aerosol effects on forests

Plants use diffuse radiation, scattered by aerosol particles, more efficiently than direct radiation. This ‘diffuse fertilisation effect’ (DFE) results from a more even distribution of diffuse radiation within the canopy, and photosynthesis increases with absorbed radiation, but at a diminishing rate. Thus, canopies gain more carbon when many leaves receive moderate light than when a few leaves are highly illuminated. Diurnal dynamics of DFE depend on leaf position: mid‐ to lower‐canopy leaves, normally shaded under clear conditions, benefit from DFE throughout the day, while upper‐canopy leaves benefit from DFE at midday, by preventing midday stomatal closure (Z. Wang *et al*., 2022). Aerosol deposition can also affect stomatal conductance and optical properties, although modestly (Grantz *et al*., 2018).

A key question is whether the benefits of increased diffuse fraction overcome the dimming effect on the total amount of radiation caused by aerosol particles and clouds (see also Box 2). Ecosystem photosynthesis generally peaks at a diffuse fraction of 0.4–0.6 (see Zhou *et al*., 2021), typical of thin or patchy clouds (van Diepen *et al*., 2025). Aerosol alone rarely raises diffuse fraction above 0.2–0.3 in boreal forests (Ezhova *et al*., 2018), although high aerosol loading with cumulus clouds can enhance photosynthesis (Ezhova *et al*., 2025). At higher diffuse fractions, the dimming effect dominates and photosynthesis declines, suggesting that the absolute amount of diffuse radiation may better capture photosynthetic responses, as it combines the dimming and diffuse fraction effect in a single variable (Neimane‐Šroma *et al*., 2024). Although BSOAs primarily scatter radiation, black carbon primarily absorbs radiation. Increased black carbon emissions, for example from wildfires, could thus tip the balance between positive and negative effects of aerosols on forests (Box 1). Moreover, forest structure, such as leaf area, foliage clumping, and species, also modulates DFE. Stronger DFE generally occurs at high leaf area (Zhou *et al*., 2021), in evergreen needleleaf species, and when the vapour pressure deficit is below 5 hPa, and the air temperature is between 20 and 25°C (Gui *et al*., 2021), because stomatal closure will be the limiting factor for photosynthesis in more extreme conditions.

**Fig. 2:**
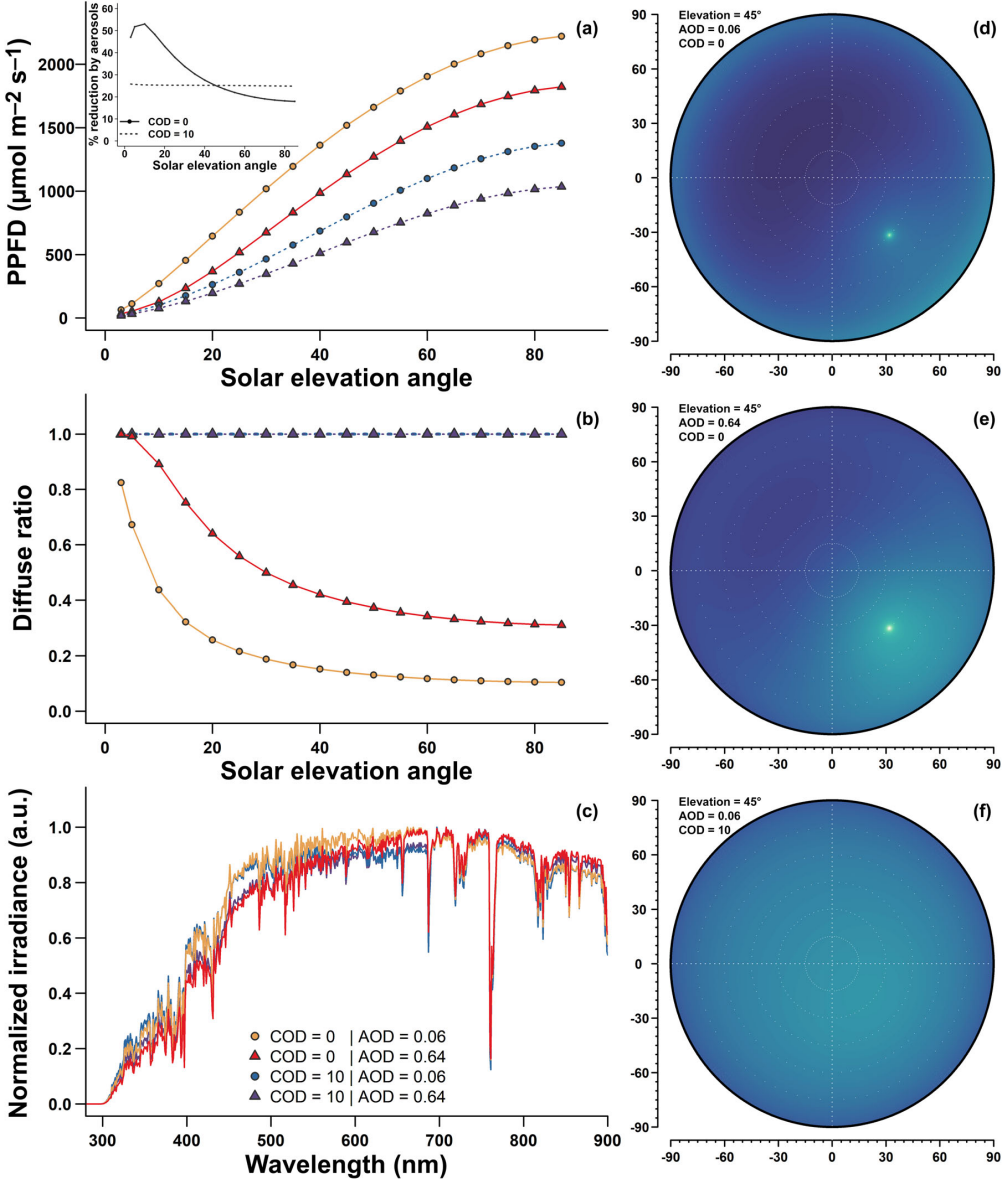
Properties of incident solar radiation as modified by aerosols and clouds. We consider four combinations of clear (cloud optical depth COD = 0) and overcast (COD = 10) sky with aerosol loading typical of a remote boreal forest (aerosol optical depth at 500 nm: AOD = 0.06, referred to as low aerosol) and of an urban site (AOD = 0.64, referred to as high aerosol). (a–c) Photosynthetic photon flux density (PPFD, a) and diffuse ratio (b) as a function of solar elevation angle and normalised spectral composition (c) under a clear sky with low aerosol loading (in yellow), a clear sky with high aerosol (in red), an overcast sky with low aerosol (in blue), or an overcast sky with high aerosol (in purple). (d–f) Sky map of diffuse radiances under a clear sky with low (d) or high aerosol (e) and an overcast sky with low aerosol (f). All data are outputs from the libRadtran (v.2.0.5) radiative transfer model using the solver ‘disort’ with pseudospherical correction, and REPTRAN in coarse mode for spectral calculations. Simulations were done over the range 250–900 nm, at altitude 0 m, pressure 1000 hPa, surface temperature 15°C, with atmospheric concentration of H_2_O as 10 kg m^−2^, O_3_ at 320 Dobson units, and NO_2_ at 0.2 Dobson units. The albedo was set as ‘evergreen needle forests’ from a collection of spectral albedos in the built‐in International Geosphere Biosphere Programme library (Loveland & Belward, 1997) and aerosols were defined by the OPAC library (Hess *et al*., 1998) as mixtures of water (in‐)soluble particles, soot, and sea salt particles set as ‘continental clean’ (AOD = 0.06) or ‘urban’ (AOD = 0.64). Date and location were not specified to manually define solar elevation angles without correcting for seasonal changes in the Earth–Sun distance.

Aerosol particles affect not only the forest carbon budget but also the water cycle. On the one hand, forest transpiration increases due to diffuse radiation that promotes stomatal opening (Pedruzo‐Bagazgoitia *et al*., 2017). On the other hand, the decline in total radiation could decrease evaporative demand and thus evapotranspiration (B. Wang *et al*., 2022). The lower proportion of incident blue light under increased aerosol (Fig. 2) can also negatively affect stomatal opening and hydraulic conductivity (Sellin *et al*., 2011; Matthews *et al*., 2020). The net effect of aerosol particles on evapotranspiration depends on the amount of diffuse fraction, whereby a decline occurs at high diffuse fraction, which can be reached under fire plumes or in urban conditions (B. Wang *et al*., 2022). The increase in aerosol load therefore further leads to an increase in photosynthetic water‐use efficiency (WUE, i.e. less water is needed to fix a unit of carbon in photosynthesis) up to an optimum: the aerosol‐driven increase in WUE is highest under moderate diffuse fraction (Zhang *et al*., 2023) where the rise in photosynthesis dominates the change in WUE (Wang *et al*., 2023). Aerosol particles also decrease surface temperatures (Zhou *et al*., 2021), both directly by reducing shortwave radiation reaching the surface and indirectly by increasing evaporative cooling (Chakraborty *et al*., 2021). Lower surface temperatures can affect forest carbon sinks both via decreased respiration and photosynthesis (Steiner & Chameides, 2005). Depending on the climatic region, which effect dominates can change, as photosynthesis and growth are temperature‐limited in high‐latitude and high‐altitude ecosystems. However, increased vapour pressure deficit from less precipitation under aerosols (Zhou *et al*., 2021) will likely have a negative impact on tree growth and survival in most climatic regions by intensifying drought stress.

## IV. Long‐term aerosol effects on forests

A key omission in global climate models evaluating aerosol impacts is the long‐term feedback on plant development (Durand *et al*., 2021), largely due to limited research in forests and plant canopies. ORCHIDEE‐DF simulations suggest the diffuse radiation fraction has risen by nearly 5% since preindustrial times (≈ 5 W m^−2^; Zhang *et al*., 2021), while CMIP6 models show a 1.01–1.11 W m^−2^ decrease in total shortwave radiation (Kalisoras *et al*., 2024). Aerosol‐driven changes in irradiance (strength, diffuseness, and spectrum) affect leaf morphology (Li *et al*., 2014a), plant architecture (Li *et al*., 2014b; González *et al*., 2019), disease and pest damage (Merfield *et al*., 2019; Fountain *et al*., 2020), and herbivory (Hakimara & Despland, 2024), with potential long‐lasting effects on forest carbon fluxes (Emmel *et al*., 2020) and community composition (Wakatsuki *et al*., 2021).

Few studies on shading effects use levels consistent with realistic aerosol‐driven reductions in light (e.g. González *et al*., 2019; Shang *et al*., 2024), or isolate shading from increases in diffuse radiation (e.g. Shao *et al*., 2019). During the global dimming period (1950–1980), aerosol‐driven reductions led to *c*. 10% less annual radiation over Europe (Wild *et al*., 2021), equivalent to 2–4 mol m^−2^ d^−1^. Such changes likely cause only minor morphological and architectural changes (see fig. 2 in Poorter *et al*., 2019). For instance, a study using realistic shading expected from aerosols (−13%) in *Vicia faba* (L.) had no effect on biomass or structure (Ryel *et al*., 2010). While stronger effects may occur in more light‐limited environments (Stanhill & Cohen, 2001), they are likely far less than the 26.5% growth reduction seen in *Oryza sativa* (L.) under 40% shading (Shang *et al*., 2024).

In the long term, diffuse radiation alters resource allocation and canopy architecture. In tomato (*Solanum lycopersicum* L.), it reduces vertical gradients of nitrogen and Chl, increases leaf mass per area and mesophyll thickness (Li *et al*., 2014a), congruent with a shift in light distribution from the upper to the lower canopy. Diffuse light also results in more compact plants with greater leaf area, more stems, and shorter height (Li *et al*., 2014b). However, no similar experiments have been conducted in trees or in forests. If diffuse radiation reduces light competition, trees may invest more in deeper canopy leaves rather than in top growth. Still, the long‐term impact of changes in diffuse radiation on forest dynamics, especially in mixed forests with species of differing light demands, remains unclear.

These long‐term considerations, added to the complex interacting processes at play in the atmosphere and in the canopy, make it exceedingly difficult to appreciate how much plant functions will be affected through future changes in radiation via aerosols. A recent estimation, missing part of the story, reports that aerosols (anthropogenic and natural) increase global gross primary productivity (GPP) nearly 1 PgC yr^−1^, or *c*. 1% of the land sink (Zhou *et al*., 2022). In practical terms, this means that doubling anthropogenic aerosols would yield only a few percent change in global carbon uptake. However, this calculation hides strong regional effects. In South‐East Asia and China, present‐day aerosol optical depth (AOD ≥ 0.8) often exceeds the optimum for diffuse gain, so further pollution will suppress GPP (Yue & Unger, 2017). Arid regions normally have near‐zero clouds and low background aerosol load (AOD ≈ 0.4), but their current level reduces productivity by 14%, because they induce fewer precipitations and total light reduction dominates over diffuse‐light gains in their largely open canopies (Evans *et al*., 2019). By contrast, boreal forests are very sensitive to aerosol load, because their relative pollution‐free atmosphere (AOD ≈ 0.06) means a small absolute rise in aerosol mass can easily double it and translate into tens of percent more diffuse radiation flux and corresponding increases in GPP by 6–14% (Ezhova *et al*., 2018, 2025). In short, though aerosols exert only modest effects on global GPP overall, their regional impacts are much stronger.

Box 2 **Direct and diffuse radiation is not a binary paradigm**

Although clouds, aerosol particles, and molecules in the atmosphere scatter sunlight, thereby producing diffuse radiation, the resulting radiation has a very different spectral distribution (Fig. 2). The primarily forward scattering of radiation by aerosol (Mie scattering) means that the photon trajectory is only weakly affected during scattering. Therefore, the diffuse radiation produced by aerosols is much less isotropic than that produced by smaller atmospheric elements (Rayleigh scattering). Thus, in terms of the direction of radiation, the diffuse light produced in an aerosol‐loaded atmosphere under a cloud‐free sky will be much more similar to the conditions of a clear sky without aerosol than to a cloudy or overcast sky (Fig. 2a–c). The resulting radiation distribution within canopies and leaves will likely be intermediate between the incident radiation under a clear sky and total diffuse radiation produced by clouds. In essence, if all scattered (diffuse) radiation conserved an origin within the solar disc when incident on forests, their effect compared to direct radiation would be limited only to the effects resulting from the modification of the spectral composition. This also means that proportionally more radiation is lost to space when the sun is low in the sky because of a ‘ricochet’ effect (Fig. 2d; Wild *et al*., 2021).

This stronger diurnal and seasonal effect on radiation reduction by aerosol could affect spring phenology and autumn senescence, as they both depend strongly on photoperiod in many species (Richardson *et al*., 2013). Unlike the incident radiation under clouds, which is enriched in blue and ultraviolet (UV) radiation, aerosols tend to have the opposite effect of depleting the incident radiation in these shorter wavelengths (Fig. 2e; Durand *et al*., 2021). This is especially true for aerosols resulting from biomass burning and wildfire (Dubovik *et al*., 2002). Plants growing under reduced UV and blue radiation also tend to produce a shade avoidance response, promoting growth via increased height and leaf area but reduced leaf mass per area (González *et al*., 2019). These differences are also known to affect leaf functions (Brodersen & Vogelmann, 2010). Thus, care is needed to account for these effects when designing experiments assessing the effect of aerosols on plants.

## V. Conclusion

We still know little about aerosol effects on plants and forests, both in the short‐ and in the long‐term. This is partly due to a few detailed, co‐located *in situ* measurements of ecosystem–atmosphere interactions, such as station for measuring ecosystem‐atmosphere relations (SMEAR) (Hari & Kulmala, 2005) and Amazon Tall Tower Observatory (ATTO) (Andreae *et al*., 2015) for boreal and tropical forests, respectively. As aerosol particles have several indirect effects on vegetation via radiation, temperature, clouds, and water availability, it is challenging to capture causality in dynamic field conditions. Thus, more controlled experimental frameworks designed to also assess long‐term effects are critically needed. Using filters with realistic levels of shading and diffuseness (e.g. Ryel *et al*., 2010) will yield precious information on short‐term physiological effects that can be integrated into photosynthesis submodels, as well as long‐term morphological changes of leaf and canopy structure. Extensive mapping of direct and diffuse radiation, temperature, and humidity is now within grasp using low‐cost sensors. They could help bridge the gap between more expansive (and expensive) stations measuring surface energy, water, carbon budget, and atmospheric processes, which remain rare but crucially needed, especially in dense tropical forests.

Prediction variation between models at the same aerosol loading can exceed the overall effect of aerosols within a single model (Chakraborty & Lee, 2021), partly because they treat diffuse radiation differently. Extended and integrated model intercomparisons (e.g. RAMI, CMIP and GeoMIP) will help assess the uncertainties built into different models. Beyond this, improved confidence in predictions will necessitate further development of mechanistic or AI‐based models of atmospheric chemistry, SOA formation, aerosol–cloud interactions, and their effect on solar radiation and precipitation, as well as plant community dynamics. At the canopy level, only a few ecosystem models reproduce the DFE, and none include the under‐researched effect at the leaf level (Berry & Goldsmith, 2020). Advancing our understanding of aerosol–forest interactions, integrating key mechanisms into models, and validating them with global observations is essential to disperse this haze of uncertainty and produce better evidence‐based policies.

## Competing interests

None declared.

## Author contributions

MD, AL and EE contributed equally to the conceptualisation of the review and the writing of the main text. MD collected the data illustrated in Fig. 2, wrote the abstract, introduction, and boxes.

## Disclaimer

The New Phytologist Foundation remains neutral with regard to jurisdictional claims in maps and in any institutional affiliations.

## Supporting information


**Table S1** Dataset used for Fig. 2.
Please note: Wiley is not responsible for the content or functionality of any Supporting Information supplied by the authors. Any queries (other than missing material) should be directed to the *New Phytologist* Central Office.

## Data Availability

The data supporting the findings of this study are available in the Supporting Information of this article (Table S1).
